# Minimally Invasive Surgery for Perihilar Cholangiocarcinoma—A Review of the Current Literature

**DOI:** 10.3390/jcm14113748

**Published:** 2025-05-27

**Authors:** Panagiotis Dorovinis, Nikolaos Machairas, Alexios Terra, Ifaistion Palios, Stylianos Kykalos, Dimitrios Dimitroulis

**Affiliations:** 1 2nd Department of Propaedeutic Surgery, Laiko General Hospital, National and Kapodistrian University of Athens Medical School, 115 27 Athens, Greece; nmachair@gmail.com (N.M.);; 2 Colorectal Cancer Unit, Norfolk and Norwich University Hospital, Norwich NR4 7UY, UK; 3 Department of Transplantation Surgery, Ippokrateio General Hospital, Aristotle University of Thessaloniki, 546 42 Thessaloniki, Greece; 4 Hellenic Minimally Invasive and Robotic Surgery M.I.R.S. Study Group, National and Kapodistrian University of Athens Medical School, 115 27 Athens, Greece

**Keywords:** perihilar cholangiocarcinoma, hilar cholangiocarcinoma, Klatskin tumor, robotic, laparoscopic, minimally invasive surgery

## Abstract

Perihilar cholangiocarcinoma (pCCA) is the most common subtype of a rare malignancy arising from the biliary tract. Its challenging diagnosis results in delayed treatment, most often when the disease is locally advanced or widespread. Management includes surgery followed by systemic chemotherapy; however, a negative resection margin (R0) is the mainstay for achieving an adequate survival benefit, in the absence of metastatic disease. While minimally invasive surgery (MIS) initially is adopted across every surgical field, laparoscopy’s inherent limitations hinder its implementation for the treatment of pCCA and results in skepticism even for the robotic approach. However, since its initial feasibility phase fifteen years ago, comparable results to open surgery have been reported regarding its safety and oncologic outcomes, in highly selected patients. Moreover, the robotic approach seems to be associated with favorable outcomes regarding post-operative complications, length of hospital stay, and estimated blood loss. International guidelines for the diagnosis and management of pCCA, centralization, definition of a learning curve for MIS, and more comparative studies assessing long term outcomes and randomization are key elements to ensure patient’s safety and technical efficiency. The aim of our review is to provide an updated perspective of the existing literature in the utilization of MIS for patients with pCCA.

## 1. Introduction

Cholangiocarcinomas (CCAs) represent a heterogenous group of malignancies originating from epithelial cells of the biliary tract. Although they are the second most common primary hepatic malignancy following hepatocellular carcinoma (HCC), they represent 15% of all primary hepatic malignancies and only 3% of the malignancies of the gastrointestinal tract [1]. They can arise either from the intrahepatic and extrahepatic bile ducts and can be divided further into intrahepatic (iCCA), perihilar (pCCA), and distal (dCCA) cholangiocarcinoma according to their exact anatomic location [2]. Although they represent a rare clinical entity with an incidence of 0.3 to 6 cases per 100,000 inhabitants per year, they are associated with an overall five-year survival rate not exceeding 20% and a high recurrence rate, resulting in a poor overall prognosis [3]. Additionally, patients develop painless jaundice, which is the most common symptom, late over the course of the disease, when the primary tumor is most likely to be locally advanced [4].

In particular, perihilar cholangiocarcinomas refer to tumors arising between the second order bile ducts and the cystic duct and have been characterized by distinctive clinical and pathological characteristics. They represent the most common subgroup, account for 50–60% of all cholangiocarcinoma types and are distinguished by a unique tendency to invade adjacent vascular structures [5]. Complete surgical resection (R0), followed by systemic chemotherapy, remains the mainstay for the treatment of pCCA, offering the highest probability of long-term survival [3,6]. However, its late diagnosis in conjunction with the invasion of proximal vascular structures hinders resection, making it one of the most technically demanding operations commonly associated with biliary and vascular reconstructions.

Another important prognostic factor for survival is lymph-node metastasis. In a 2022 study, Nooijen et al. concluded that in the presence of positive regional lymph nodes, overall survival (OS) and disease-free survival (DFS) were not determined by whether an R0 resection was achieved or not. Therefore, an adequate lymph node sampling to diagnose the infiltration of the regional lymph nodes would be beneficial to the patient [7].

Hence, in the absence of widespread disease, R0 resection constitutes a prerequisite for long-term survival. Several techniques have been suggested to that aim, including resection of the caudate lobe, venous and arterial resections, the “non-touch” technique and even transplantation in highly selected patients [8,9]. Furthermore, minimally invasive surgery (MIS), which has been implemented in almost every surgical field and is associated with several bestowed advantages, has been used by experienced minimally invasive hepatobiliary surgeons in the resection of perihilar cholangiocarcinoma. However, the technically demanding nature of pCCA resection and some inherent limitations of the laparoscopic approach, specifically, have resulted in a reluctancy regarding its equal oncologic outcomes [10]. This delayed the wide adoption of MIS, until Giulianotti et al. reported a robot-assisted laparoscopic extended right hepatectomy with biliary reconstruction for a patient with pCCA, in 2010 [11]. Despite the recent technical and pharmacological advances, the diagnosis and treatment of these patients remains challenging and they are most successfully managed within a multidisciplinary team, which was proved almost a decade ago [12]. The aim of our review is to provide an updated perspective of the existing literature for the utilization of MIS for the treatment of patients with pCCA.

## 2. Materials and Methods

A review of the current literature was undertaken in Medline (PubMed) with the objective of identifying studies reporting outcomes regarding open and minimally invasive resections for perihilar cholangiocarcinoma. The terms utilized included: “perihilar cholangiocarcinoma”, “hilar cholangiocarcinoma”, “Klatskin tumor”, “robotic”, “laparoscopic”, “minimally invasive surgery” combined with the Boolean operators AND/OR. Only articles published in English until December 2024 were considered. Our research generated six case series, with the majority from single centers with extensive experience in MIS cholangiocarcinoma resection, five metanalysis and systematic reviews, data from a single registry, one case report, two multicenter studies and two comparative analyses. Although we opted not to follow formal guidelines for article selection, we chose either large case series or comparative studies.

## 3. Results

### 3.1. Open vs. Minimally Invasive Surgery

Surgical resection through the open approach has been the mainstay for the treatment of resectable perihilar cholangiocarcinoma. Direct visualization of the hepatic hilum allows for the delicate manipulation of critical anatomical structures (portal vein, hepatic artery, bile duct). Moreover, tactile sensation can guide surgical actions more precisely in the presence of infiltration of key vascular structures and aid in the identification and removal of lymph nodes necessary for the accurate staging and treatment of the disease. Another key aspect of open surgery may be the more effective management of intraoperative adverse events, such as bleeding, that require fast and precise actions, without a potential delay, e.g., camera cleaning, changing of the instruments, conversion to open surgery. Additionally, liver resection for perihilar cholangiocarcinoma is associated with complex dissection in the porta hepatis and the requirement of biliary reconstruction is traditionally more effectively conducted through an open approach. On the other hand, minimally invasive procedures, in general, are associated with less trauma, reduced postoperative pain, improved cosmetic results and faster recovery. Other advantages include a magnified visual field, better exposure of key anatomical structures, e.g., caudal to cephalad vision of the short hepatic veins, enhanced precision and manual dexterity in tight areas and improved ergonomics for surgeons without compromising the patients’ oncologic outcome when performed by trained surgeons in expert centers. Moreover, the use of ICG during minimally invasive surgery can aid in the identification of the key anatomical structures of the hepatic hilum and define the dissection plane during hepatectomy. However, the main downside of the minimally invasive approaches is that, in general, they are more time consuming, requiring prolonged docking time.

### 3.2. Laparoscopic Resection of Perihilar Cholangiocarcinoma

The current advancements in minimally invasive surgical techniques have inevitably infiltrated the field of hepatopancreatobiliary (HPB) surgery. Laparoscopic liver and pancreatic resections have proven to be safe and efficient alternatives resulting in outcomes comparable to open resection [13]. In a metanalysis of laparoscopic versus open hepatic resection from 2018, Jin et al. demonstrated that laparoscopy is superior to the open approach regarding the length of stay (LOS), estimated blood loss (EBL) and complication rate [14]. These results are of great significance, since laparoscopic resection of pCCAs requires biliary resection and reconstruction, with the concurrent likelihood of vascular reconstruction in a complex anatomic area in close proximity to both the pancreas and the vascular structures of the hepatic hilum. In a recent multicenter analysis among 645 patients from China, the laparoscopic approach was associated with shorter LOS (mean 14.32 vs. 17.95 d, *p* < 0.001) and a lower severe complication rate (CD ≥ III) (12.11% vs. 22.88%, *p* = 0.006) [15]. In another retrospective analysis, He et al. demonstrated significantly less EBL [laparoscopic: 400–800 mL vs. open: 200–400 mL (*p* = 0.012)] in the laparoscopic group, as well as longer operative time (OT) [laparoscopic: (407.97 ± 76.06) min vs. open: (489.69 ± 79.17) min (*p* = 0.001)], with no significant difference in post-operative complications [16]. A retrospective survival analysis from Yin et al. concluded that laparoscopic pCCA resection offers the same clinical efficacy compared to open resection [17]. In a single center cohort study by Xiong et al., laparoscopic resection for pCCA was found to be similar to open resection, in terms of safety and radicality, with the rational trocar distribution and total caudate lobectomy attributing to the benefits of this approach. Additionally, the caudal to cephalad approach allows for better visualization of segment I, providing an additional technical benefit for its removal, facilitating the oncologic adequacy of laparoscopy. In a recent systematic review of fifteen studies by Berardi et al., a negative resection margin (R0) was obtained in 82.4% of patients, minor and major morbidity events occurred in 41.3% and 12.7% of the included patients, respectively, and the conversion rate was 11.6% most likely attributed to the challenging bile duct reconstructions required [18].

In another comparative analysis of 2020, Ratti et al. conducted a propensity score matching analysis between 16 patients undergoing a laparoscopic resection for perihilar cholangiocarcinoma. They demonstrated statistically significant longer OT (360 vs. 275 min, *p* = 0.048), less EBL (380 vs. 470 mL, *p* = 0.048), less intraoperative blood transfusion 12.5% vs. 21.9%, *p* = 0.032) and a shorter LOS (10 vs. 14 days, *p* = 0.048). The number of harvested lymph nodes and the R0 resection rate showed no significant differences between the two groups [19]. However, a key point is that in this study, the biliary-enteric anastomosis was conducted through the service incision [Table 1].

### 3.3. Robotic Resection of Perihilar Cholangiocarcinoma

In an effort to reduce morbidity from open liver resections, Professor P.C. Giulianotti performed the first robot-assisted laparoscopic extended right hepatectomy with biliary reconstruction in 2010 [11]. This paved the way for the implementation of this novel technique by many centers worldwide. In an initial case series of 10 patients, published in 2016, Xu et al. demonstrated increased OT (703 ± 62 vs. 475 ± 121 min, *p* < 0.001), higher morbidity [90 (9/10) vs. 50%, *p* = 0.031] and greater hospital expenditures (27,427 ± 21,316$ vs. 15,282 ± 5957$, *p* = 0.018). This led the authors to conclude that, despite its technical feasibility, the robotic approach for cholangiocarcinoma should not be performed, unless technical improvements arise [20]. Those two pioneering attempts were embraced with great enthusiasm by the scientific community, leading to an increasing number of experienced surgeons in high-volume centers, implementing this approach in selected patients. Cillo et al. reported four initial cases of left hepatectomy with caudate lobectomy for patients with perihilar cholangiocarcinoma. Three patients developed minor complications, no major complications were evident, and one patient developed a biliary leak, managed cautiously. One of the cases was converted to open surgery, and in one of the patients the biliary margin was positive [21]. Magistri et al. demonstrated the results of a case series of fourteen patients having undergone robotic resection for perihilar cholangiocarcinoma. These have been a median EBL of 150 mL (range 50–800 mL), a median OT of 490 min (range 390–750 min) and a median LOS of 6 days (range 3–91). R0 resection rate was 92.9%, median number of lymph nodes retrieved was 19 (range 11–40), there was no 90-day mortality reported and 3-year survival exceeded 50%, supporting not only the feasibility of this approach but also its safety [22]. The first international multicenter prospective analysis of robotic resection for perihilar cholangiocarcinoma by Sucandy et al. included 38 patients, out of whom three underwent vascular reconstruction and 18% had >1 biliary anastomosis. The short-term outcomes were a median of 200 mL EBL, a median OT of 481 min, a median harvested lymph nodes 7, and R0 margins were achieved in 82% of patients. Major complications were evident in 16% of patients, LOS was 6 days and there was 1 post-operative death reported within 30 days. Regarding long-term outcomes, with a median follow-up of 15 months, overall survival was 71%, and disease-free survival was 68% [23]. Finally, Christodoulou et al. conducted a recent analysis of short-term outcomes and survival for patients with biliary tract cancers treated by utilizing the robotic platform. They prospectively followed 27 patients undergoing robotic surgical resection for perihilar cholangiocarcinoma, 22 out of whom required a concomitant hepatectomy. Regarding operative outcomes, the median OT was 470 min (422 ± 111 min), the median EBL was 150 mL (171 ± 123 mL), the mean number of harvested lymph nodes was 5 ± 2.7 and a negative resection margin was achieved in 85% of patients. During the post-operative period, three patients developed minor complications and two developed major complications, with one patient requiring reoperation due to an incarcerated hernia and one 90-year-old patient developing emphysema and dying from respiratory failure within 90 days. Within a median follow-up time of 27 (27 ± 17) months, disease-free survival was 70% and overall survival was 88% [24] [Table 2].

## 4. Discussion

In the era of robotic surgery applied in a vast number of HPB diseases and even liver transplantation for selected patients, it seems that there is a scarcity of published data regarding minimally invasive resections for perihilar cholangiocarcinoma. In one of the initial publications on the topic, Xu et al. concluded that robotic resection for perihilar cholangiocarcinoma, though feasible when performed by experienced surgeons, was not fully supported until significant technical improvements were evident [20]. In addition, it was suggested by the majority of the reported literature that minimally invasive techniques for the treatment of this clinical entity are reserved for highly selected patients, high volume centers and experienced HPB surgeons, associated with a steep learning curve. Adding up to the former, the rarity of the tumor itself and its tendency to develop symptoms late over the course of the disease, not until locally advanced, translates into less patients benefiting from the approach and less surgeons being able to perform such an operation.

The technical limitations of the laparoscopic instruments, characterized by a narrow range and limited axis of motion, have created a steep learning curve, since the required delicate manipulation of the structures in the hepatic hilum and the need for suturing and reconstructions in tight spaces constitute an inherent disadvantage of laparoscopy. This is reflected upon the higher conversion rate when compared to the robotic approach. Nevertheless, aside from its technical feasibility, the oncologic adequacy of this approach is yet to be confirmed.

The first reported case series of laparoscopic resection for perihilar cholangiocarcinoma has been reported by Yu et al. in 2011 [25]. This included fourteen patients with Bismuth type I and II perihilar cholangiocarcinoma [25]. One year prior to this publication, Giulianotti et al. reported the first case of robot-assisted laparoscopic extended right hepatectomy with biliary reconstruction for a patient with perihilar cholangiocarcinoma [11]. These initial reports paved the way for an increasing number of publications regarding minimally invasive surgery for perihilar cholangiocarcinoma. However, the robotic platform is associated with many bestowed advantages over laparoscopy, such as a significantly improved three-dimensional field of view, a superior range of motion of the robotic arms, tremor elimination and the ability for refined movements and manipulation of the tissues. These advantages are clearly emphasized in the operative management of perihilar cholangiocarcinoma, since the delicate manipulation of the perihilar structures is a prerequisite for the safe performance of the procedure and for the adequate oncologic outcome of the patient, e.g., complex resection, reconstruction and mandatory lymphadenectomy. This shift is reflected upon the growing number of published literatures from centers implementing a robotic approach. Despite this wide adoption, the long-term oncologic outcomes of minimally invasive surgery (MIS) for pCCA remain insufficiently defined. Randomized controlled trials lack the reporting of this evidence, which also leads to a lack of the optimal criteria for patient selection and the standardization of the procedure. Furthermore, cross-institutional comparisons are hindered by global economic disparities, which along with the variable access to technological advancements, makes randomization inefficient or even misleading.

Perihilar cholangiocarcinoma is a rare disease with a subclinical presentation in the early stages, hindering definite diagnosis even when there is strong clinical suspicion. Recent advances in the field of liquid biopsy could potentially address this issue. Biomarkers, including circulating tumor cells, cell-free DNA, circulating tumor DNA, RNA and extracellular vesicles can be detected in peripheral blood samples from patients with high suspicion of pCCA. Moreover, cholangiocarcinoma is characterized by a high mutational burden, offering a broader array of genetic targets for early detection. Advancements in deep learning could also potentially assist with the early detection of cholangiocarcinoma. Both in the interpretation of endoscopic ultrasound-derived images [26], as well as in the analysis of complex imaging and interpretation of clinical data, machine learning models have proved their efficiency both for the distal as well as the intrahepatic cholangiocarcinoma. This could be expanded to facilitate an earlier and definite diagnosis for patients with pCCA also.

Furthermore, it is difficult to find patients in whom minimally invasive resection would be feasible and beneficial. This is reflected upon the most recent study by Christodoulou et al. [24], originating from a center with great experience in robotic HPB surgery. They conducted a prospective 10-year study, during which they managed to recruit 27 patients that underwent robotic-assisted biliary resection for perihilar cholangiocarcinoma with or without contaminant hepatectomy. Therefore, we can easily conclude that to overcome the learning curve of the procedure, it requires a long time period, and it is conducted in selected patients, even in expert centers.

In spite of the aforementioned advantages of a robotic approach for perihilar cholangiocarcinoma and its superiority over laparoscopy or open surgery, waiting to be established in selected cases, its widespread adoption is still limited. Many factors contribute to this phenomenon. The prohibitive cost of obtaining and operating a robotic system in low- and middle-income economies, the complex nature of the disease itself, its low prevalence, and the lack of experienced surgeons constitute a vicious cycle between the need for high patient selection and a high learning curve for the operating surgeon.

The inherent limitations of surgical research, such as the lack of randomization and the majority of the studies being retrospective analyses, associated with selection bias, applies to this review. Only two prospective studies were retrieved from our search and only a few were comparative analyses. Even on the larger retrospective series, the data on oncologic outcomes were limited. Most studies provided information on the number of lymph nodes retrieved and the percentage of R0 resections in order to support the oncologic efficacy of the approach. However, disease free and overall survival, which are the most important outcomes to establish the safety and efficacy of an operation, measured for a limited follow-up time. This is further complicated by the different pre- and post-operative management of patients with perihilar cholangiocarcinomas, resembled in the different approaches between eastern and western HPB centers, where, for example, the biliary drainage is conducted via nasobiliary drainage, in the east, or percutaneous transhepatic biliary drainage, in the west. Furthermore, the economic disparities worldwide hinder the access to laparoscopic or robotic systems (or make them unavailable 24/7), interventional radiology or modern diagnostic procedures, e.g., cholangioscopy, result in bias when submitting cases in international registries and multicenter studies. Global registries should be strengthened and supported across all centers, regardless of volume. However, when interpreting outcomes derived from these registries, careful consideration must be given to subgroup analyses, including stratification by Gini coefficient and Human Development Index (HDI), to account for socioeconomic and systemic disparities. These inherent limitations should be considered when interpreting results from multicenter studies.

## 5. Conclusions

In particular, minimally invasive techniques and robotic surgery have been progressively integrated in many complex operations, since they are associated with technical advantages and improved patient outcomes. However, the most valuable tool when treating patients with such a difficult disease as perihilar cholangiocarcinoma remains a multidisciplinary approach. The input of the different specialties will help analyze the data derived from modern diagnostic techniques, such as liquid biopsy and machine learning algorithms interpreting data derived from endoscopic or radiologic images, in order to select patients that would benefit the most from a minimally invasive approach. This is emphasized by the lack of a widely standardized selection process and global consensus. Moreover, it is “sine qua non”, that such procedures should be undertaken in high volume centers, by experienced surgeons in both open and minimally invasive HPB surgery, to ensure the adequacy of the procedure and the safety of the patient.

## Figures and Tables

**Table 1:** Studies including data of laparoscopic resection of pCCA.

Study	R0 Resection Rate % (Lap vs. Open)	EBL (mL) (Lap vs. Open)	LOS (Days) (Lap vs. Open)	Complication Rate (%) (Lap vs. Open)	Mortality (%) (Lap vs. Open)
Jin et al. (2018) [14]	Not specified	MD over open = −164.31 mL, 95%CI: −220.91 to −107.72, *p* < 0.0001, I^2^ = 98%	MD over open = −3.84 days, 95%CI: −5.05 to −2.63, *p* < 0.0001, I^2^ = 88%	RR over open = 0.29, 95%CI: 0.17–0.50, *p* < 0.0001, I^2^ = 0%, absolute 13 to 40 fewer	Not specified
Wang et al. (2023) [15]	85.1 (lap) vs. 87.6 (open), *p* = 0.36	Median: 200 (lap) vs. 300 (open), *p* = 0.685	14.32 (lap) vs. 17.95 (open), *p* < 0.001	12.11 (lap) vs. 22.88 (open), *p* = 0.006	3.1 (lap) vs. 6.1 (open), *p* = 0.082
He et al. (2022) [16]	93.7 (lap) vs. 87.5 (open), *p* = 0.86	Median: 300 (lap) vs. 600 (open), *p* = 0.012	Median: 11.5 (lap) vs. 14 (open), *p* = 0.254	12.5 (lap) vs. 21.8 (open), *p* = 0.695	1 (lap) vs. 0 (open), *p* = 0.721
Yin et al. (2024) [17]	80 (lap) vs. 78.6 (open), *p* = 0.55	350 (lap) vs. 550 (open), *p* = 0.062	17 (lap) vs. 19 (open), *p* = 0.027	28 (lap) vs. 21 (open), *p* = 0.786	7 (lap) vs. 2 (open), *p* = 0.289
Berardi et al. (2023) [18]	82.4 (lap)	Range: 101.1 ± 13.6 to 1360 ± 809 (lap)	Range: 5.9 ± 2.1 to 36.2 ± 9.5 days (lap)	41.3% (minor), 12.7% (major) (lap)	Not specified
Ratti et al. (2020) [19]	81.3 (lap) vs. 53.1 (open)	380 (lap) vs. 470 (open), *p* = 0.048	10 (lap) vs. 14 (open), *p* = 0.048	12.5 (lap) vs. 15.6 (open)	0 (lap) vs. 0 (open)

**Table 2:** Studies including data of robotic resection of pCCA.

Study	R0 Resection Rate % (Rob vs. Open)	EBL (mL) (Rob vs. Open)	LOS (Days) (Rob vs. Open)	Complication Rate (%) (Rob vs. Open)	Mortality (%) (Rob vs. Open)
Xu et al. (2016) [20]	70 (Rob)	1360 (Rob) vs. 1014 (open)	16 (Rob) vs. 14 (open)	30 (Rob) vs. 16 (open)	10 (Rob) vs. 6.3 (open)
Cillo et al. (2021) [21]	75 (Rob)	700 (Rob)	9 (Rob)	0 (Rob)	Not specified
Magistri et al. (2023) [22]	92.9 (Rob)	150 (range 50–800) (Rob)	6 (range 3–91) (Rob)	21.5 (Rob)	0 (Rob)
Sucandy et al. (2024) [23]	82 (Rob)	200 (Rob)	6 (Rob)	16 (Rob)	2.6 (Rob)
Christodoulou et al. (2024) [24]	87 (Rob)	200 (Rob)	4 (Rob)	19 (Rob)	32 (Rob)
Berardi et al. (2023) [18]	72.6 (Rob)	Range: 150–1360 (Rob)	Range: 9–16 (Rob)	12.9 (Rob)	1.6 (Rob)

## Data Availability

Data sharing is not applicable.

## References

[1] Fitzmaurice C., Dicker D., Pain A., Hamavid H., Moradi-Lakeh M., MacIntyre M.F., Allen C., Hansen G., Woodbrook R., Global Burden of Disease Cancer Collaboration (2015). The Global Burden of Cancer 2013. JAMA Oncol..

[2] Nakeeb A., Pitt H.A., Sohn T.A., Coleman J., Abrams R.A., Piantadosi S., Hruban R.H., Lillemoe K.D., Yeo C.J., Cameron J.L. (1996). Cholangiocarcinoma. A spectrum of intrahepatic, perihilar, and distal tumors. Ann. Surg..

[3] Banales J.M., Cardinale V., Carpino G., Marzioni M., Andersen J.B., Invernizzi P., Lind G.E., Folseraas T., Forbes S.J., Fouassier L. (2016). Cholangiocarcinoma: Current knowledge and future perspectives consensus statement from the European Network for the Study of Cholangiocarcinoma (ENS-CCA). Nat. Rev. Gastroenterol. Hepatol..

[4] Nagino M., Ebata T., Yokoyama Y., Igami T., Sugawara G., Takahashi Y., Nimura Y. (2013). Evolution of surgical treatment for perihilar cholangiocarcinoma: A single-center 34-year review of 574 consecutive resections. Ann. Surg..

[5] Klatskin G. (1965). Adenocarcinoma of the Hepatic Duct at Its Bifurcation within the Porta Hepatis. An Unusual Tumor with Distinctive Clinical and Pathological Features. Am. J. Med..

[6] Wang M.L., Ke Z.Y., Yin S., Liu C.H., Huang Q. (2019). The effect of adjuvant chemotherapy in resectable cholangiocarcinoma: A meta-analysis and systematic review. Hepatobiliary Pancreat. Dis. Int..

[7] Nooijen L.E., Banales J.M., de Boer M.T., Braconi C., Folseraas T., Forner A., Holowko W., Hoogwater F.J.H., Klumpen H.J., Groot Koerkamp B. (2022). Impact of Positive Lymph Nodes and Resection Margin Status on the Overall Survival of Patients with Resected Perihilar Cholangiocarcinoma: The ENSCCA Registry. Cancers.

[8] DeOliveira M.L., Cunningham S.C., Cameron J.L., Kamangar F., Winter J.M., Lillemoe K.D., Choti M.A., Yeo C.J., Schulick R.D. (2007). Cholangiocarcinoma: Thirty-one-year experience with 564 patients at a single institution. Ann. Surg..

[9] Cillo U., Fondevila C., Donadon M., Gringeri E., Mocchegiani F., Schlitt H.J., Ijzermans J.N.M., Vivarelli M., Zieniewicz K., Olde Damink S.W.M. (2019). Surgery for cholangiocarcinoma. Liver Int..

[10] Hu H.J., Wu Z.R., Jin Y.W., Ma W.J., Yang Q., Wang J.K., Liu F., Li F.Y. (2019). Minimally invasive surgery for hilar cholangiocarcinoma: State of art and future perspectives. ANZ J. Surg..

[11] Giulianotti P.C., Sbrana F., Bianco F.M., Addeo P. (2010). Robot-assisted laparoscopic extended right hepatectomy with biliary reconstruction. J. Laparoendosc. Adv. Surg. Tech. A.

[12] Gomez D., Patel P.B., Lacasia-Purroy C., Byrne C., Sturgess R.P., Palmer D., Fenwick S., Poston G.J., Malik H.Z. (2014). Impact of specialized multi-disciplinary approach and an integrated pathway on outcomes in hilar cholangiocarcinoma. Eur. J. Surg. Oncol..

[13] Nickel F., Haney C.M., Kowalewski K.F., Probst P., Limen E.F., Kalkum E., Diener M.K., Strobel O., Muller-Stich B.P., Hackert T. (2020). Laparoscopic Versus Open Pancreaticoduodenectomy: A Systematic Review and Meta-analysis of Randomized Controlled Trials. Ann. Surg..

[14] Jin B., Chen M.T., Fei Y.T., Du S.D., Mao Y.L. (2018). Safety and efficacy for laparoscopic versus open hepatectomy: A meta-analysis. Surg. Oncol..

[15] Wang M., Qin T., Zhang H., Li J., Deng X., Zhang Y., Zhao W., Fan Y., Li D., Chen X. (2023). Laparoscopic versus open surgery for perihilar cholangiocarcinoma: A multicenter propensity score analysis of short- term outcomes. BMC Cancer.

[16] He Y.G., Huang W., Ren Q., Li J., Yang F.X., Deng C.L., Li L.Q., Peng X.H., Tang Y.C., Zheng L. (2022). Comparison of Efficacy and Safety Between Laparoscopic and Open Radical Resection for Hilar Cholangiocarcinoma-A Propensity Score-Matching Analysis. Front Oncol..

[17] Yin Y., Tao J., Xian Y., Hu J., Li Y., Li Q., Xiong Y., He Y., He K., Li J. (2024). Survival analysis of laparoscopic surgery and open surgery for hilar cholangiocarcinoma: A retrospective cohort study. World J. Surg. Oncol..

[18] Berardi G., Lucarini A., Colasanti M., Mariano G., Ferretti S., Meniconi R.L., Guglielmo N., Angrisani M., Usai S., Borcea M.C. (2023). Minimally Invasive Surgery for Perihilar Cholangiocarcinoma: A Systematic Review of the Short- and Long-Term Results. Cancers.

[19] Ratti F., Fiorentini G., Cipriani F., Catena M., Paganelli M., Aldrighetti L. (2020). Perihilar cholangiocarcinoma: Are we ready to step towards minimally invasiveness?. Updates Surg..

[20] Xu Y., Wang H., Ji W., Tang M., Li H., Leng J., Meng X., Dong J. (2016). Robotic radical resection for hilar cholangiocarcinoma: Perioperative and long-term outcomes of an initial series. Surg. Endosc..

[21] Cillo U., D’Amico F.E., Furlanetto A., Perin L., Gringeri E. (2021). Robotic hepatectomy and biliary reconstruction for perihilar cholangiocarcinoma: A pioneer western case series. Updates Surg..

[22] Magistri P., Pang N.Q., Guidetti C., Caracciolo D., Odorizzi R., Catellani B., Guerrini G.P., Di Sandro S., Di Benedetto F. (2023). Robotic approach for perihilar cholangiocarcinoma: From Bismuth 1 to vascular resection. Eur. J. Surg. Oncol..

[23] Sucandy I., Marques H.P., Lippert T., Magistri P., Coelho J.S., Ross S.B., Chumbinho B., Di Sandro S., DiBenedetto F. (2024). Clinical Outcomes of Robotic Resection for Perihilar Cholangiocarcinoma: A First, Multicenter, Trans-Atlantic, Expert-Center, Collaborative Study. Ann. Surg. Oncol..

[24] Christodoulou M., Pattilachan T., Ross S.B., Rosemurgy A., Sucandy I. (2024). A single institution’s experience with robotic resections of biliary tract cancers: An analysis of the short-term outcomes and long-term survival. J. Gastrointest. Surg..

[25] Yu H., Wu S.D., Chen D.X., Zhu G. (2011). Laparoscopic resection of Bismuth type I and II hilar cholangiocarcinoma: An audit of 14 cases from two institutions. Dig. Surg..

[26] Orzan R.I., Santa D., Lorenzovici N., Zareczky T.A., Pojoga C., Agoston R., Dulf E.H., Seicean A. (2024). Deep Learning in Endoscopic Ultrasound: A Breakthrough in Detecting Distal Cholangiocarcinoma. Cancers.

